# A mixed methods exploration of the health and caregiving experiences of fathers of children with a life-limiting condition

**DOI:** 10.1177/02692163251327877

**Published:** 2025-03-25

**Authors:** Victoria Fisher, Karl Atkin, Lorna K Fraser

**Affiliations:** 1 King’s College London, London, UK; 2 Department of Sociology, University of York, York, UK

**Keywords:** Paediatrics, palliative care, qualitative

## Abstract

**Background:**

Fathers of children with a life-limiting condition are underrepresented in the literature. We know little about their experiences of caregiving, the impact of this on their health and their support needs.

**Aim:**

To explore the health and caregiving experiences of fathers of children with a life-limiting condition, both quantitatively and qualitatively.

**Design:**

A convergent mixed methods design comprised of (1) a quantitative survey and (2) semi-structured qualitative interviews prioritising the qualitative data.

**Setting/participants:**

Thirty-two fathers of children with a life-limiting condition took part in the survey. They were recruited via social media, three UK children’s hospices and one UK children’s hospital. Twelve of these fathers went on to take part in a qualitative semi-structured interview.

**Results:**

Thematic analysis resulted in three themes: (1) Everyday precarity; (2) cumulative distress; past, present and future; (3) the scope and severity of the impact of caregiving on fathers; a lack of understanding from others. In the survey, fathers reported high levels of carer strain and distress, alongside high levels of family wellbeing and positive appraisals of caregiving.

**Conclusion:**

Fathers’ extensive and overwhelming daily routines are inflexible and unstable, leading to multidimensional precarity and a sense of overwhelm. Current care provision does not address the unique and fluctuating support needs of fathers, which are linked to those of their child, and need to be understood in the context of both parenting and caregiving. A process capable of identifying and addressing fathers’ support needs to be established.

**What is already known about this topic?**

- Our understanding about the health, experiences and support needs of parent caregivers of children with a life-limiting condition mainly comes from the perspectives of mothers.
- Little is known about the impact of caregiving on the health and wellbeing of fathers.
- To ensure that all parent caregivers are supported appropriately, we must address the underrepresentation of fathers’ experiences and preferences for support in relation to their own health.

**What this paper adds**

- Caregiving should be considered in the context of individual families and relationships, that includes the consideration of parental support needs regardless of employment status or hours spent caring for their child.
- The precarity in fathers’ accounts demonstrates their constant sense of insecurity and vulnerability, which is often related to a lack of, or inconsistent, support available to enable them to care for their child.
- Fathers felt that generic psychological support was unable to address the cumulative complexities of, and fluctuations in, their own mental health across the trajectory of their child’s illness.

**Implications for practice, policy or theory**

- There is a need for support/interventions that can consider the impact of providing extremely extensive care alongside having a seriously unwell child who is going to die.
- Services need to work in ways that ensure all family members feel supported and that any assumptions relating to roles/impact of caregiving do not hinder this work.
- Further work needs to be done to establish recruitment strategies that encourage diverse participation in research, particularly to ensure the inclusion of fathers from ethnic minority groups.

## Background

As the number of children diagnosed with a life-limiting condition increases, so do the number of parents providing care for their child at home.^1^ This care is extensive and covers a vast range of responsibilities, including complex medical care such as gastrostomy and ventilation management, as well as demanding and time-consuming medication routines.^2^ Parents must adapt to the demands of caregiving following their child’s diagnosis, often needing to become experts in their child’s care.^3^ Responsibilities include overnight care and monitoring, which gives little time to rest.^4^ Providing care 24 h a day, 7 days a week, can have a profound impact on parents’ health and wellbeing. Mothers of children with a life-limiting condition are more likely to be diagnosed with anxiety, depression, heart problems and arthritis than other mothers,^5^ and much of their distress comes from ‘battles with services’ and inappropriate support and equipment.^6^

What we understand about the experiences and health of parents, is mainly founded upon the views of mothers. Existing research surrounding fathers’ perspectives demonstrate that they also experience many challenges in response to their child’s diagnosis and treatment^7^ though studies have rarely explored the impact of such on fathers’ own health nor do they ask fathers directly about their own direct experiences of healthcare. The imbalance between maternal and paternal research is partly explained by mothers being more likely to be their child’s primary caregiver.^8^ One parent will *usually* take the bulk of the caregiving, while the other goes to work, but the way in which research has approached this topic means that the caregiving contributions of the employed parent, usually fathers, can be undermined.^8^ Furthermore, some fathers will be their child’s primary caregiver but have nonetheless been largely excluded from research.^9^ As well as failing to adequately represent fathers’ experiences, this imbalance also makes it difficult to compare the experiences of mothers and fathers, which is important in considering subsequent recommendations for policy, practice and research. Therefore, the aim of this study was to explore the health and caregiving experiences of fathers of children with a life-limiting condition.

## Methodology

### Design

A convergent exploratory mixed methods design, prioritising the qualitative findings, was used comprising:

- An online cross-sectional quantitative survey advertised on social media and through UK participant identification centres (PIC’s) between November 2021 and March 2023.
- Semi-structured online qualitative interviews with fathers carried out between November 2021 and March 2023.

Each strand was implemented concurrently, whereby integration occurred at the design, sampling, data collection, data analysis and reporting levels.^10,11^ As a qualitatively led study, the qualitative component was reported with reference to the Reflexive Thematic Analysis Reporting Guidelines (RTARG).^12^

#### Setting

Three UK-based children’s hospices and one children’s hospital acted as participant identification centres for the study. Participants were also recruited via social media.

#### Participants

Fathers of children with a life-limiting condition were included if they met the criteria defined in Table 1.

**Table 1.** Inclusion criteria.

Inclusion criteria	Exclusion criteria
• were aged 18 or above; • were the father of a child with a life-limiting condition. This included biological fathers, step-fathers, adoptive fathers, and foster fathers. • had the capacity to consent to the study.	• Aged less than 18 years • Fathers whose child has died • Those who lack the capacity to participate in the study, guided by the 2005 Mental Capacity Act

#### Sample

We recruited across organisations in England and via multiple recruitment channels (hospice, hospital, social media) to ensure variations in care provision. All eligible participants were invited to participate.

#### Recruitment

Participants were recruited through the three children’s hospices, the children’s hospital and through social media (X) between November 2021 and March 2023. For recruitment via hospices and the hospital, healthcare professionals provided eligible fathers with the study information, following which they could access the survey online. For those recruited via social media, the summary study information was provided in the advert and the full study information electronically at the beginning of the survey. Contact details for the study team were included should potential participants have had any questions. At the end of the survey, if participants wanted to take part in a qualitative interview, they added their contact details and provided consent for a researcher to contact them. A researcher contacted all participants who completed a consent-to-contact form to discuss the interview component, check eligibility and book an interview if appropriate.

#### Data collection

*Survey*: The survey was based upon multi-dimensional constructs identified as important influences of caregiver outcomes in the literature,^13,14^ as well as input and testing from a Family Advisory Board (PPI) made up of parents of children with a life-limiting condition. Question formats were taken from existing validated measures, existing studies or from national surveys. The self-report questionnaire was administered via Qualtrics (Qualtrics, Provo, UT) and collected relevant demographic information including age, family structure, education, employment, household income, ethnic origin and region. Information regarding the child’s diagnosis, functioning,^15^ care needs, sex, age, age at diagnosis and any hospice support were also captured. Data on fathers’ general physical and mental health, as well as data from the Family Appraisal of Caregiving Questionnaire for Palliative Care (FACQ-PC),^16^ PROMIS Sleep Disturbance- Short Form 8a,^17^ and Health Related Quality of Life (HRQoL) using the EQ-5D-5L^18^ was captured.

*Interviews*: Qualitative interviews were conducted over Zoom using a semi-structured topic guide (Supplemental File) which was developed in the same way as above. The interviews were conducted by VF and JH, who are qualitative researchers in paediatric palliative care. The topic guide was used to ensure that the interviews addressed the key aims of the study but with flexibility for fathers to talk about their own unique experiences and perspectives of things that were important to them in the context of their own health. Notes were taken throughout the interviews and analysis. The achieved sample of 12 fathers was in line with an acceptable sample size in relation to information power.^19^ Data was monitored during collection to ensure quality, depth and specificity to the research aims. Sampling was closed when there was sufficient data to meet the aforementioned points.

#### Analysis

*Survey*: Descriptive statistics were used to summarise the demographic factors and caregiving, health, sleep and HRQoL data. We had initially planned to conduct some more advanced analyses (regression analyses) but given the final sample size were only able to provide descriptive results.

*Interviews*: Qualitative data was organised, managed, and coded in NVivo.^20^ Interviews were recorded and transcribed verbatim and checked for accuracy prior to analysis using Braun and Clarke’s reflexive thematic analysis.^12^ This approach is centralised upon the active role of the researcher, aligning with a constructivist/interpretivist positioning. There were six phases to the process of reflexive thematic analysis, though this process is iterative and flexible, rather than linear.^21^ VF read and re-read the interview transcripts, whilst listening to the audio recordings to check for any transcription errors. Written reflections were combined with those made during the interviews. VF coded the data inductively using a mixture of semantic and latent codes. Mind-maps and coding trees were useful in creating relationships between codes and developing themes, alongside frequent discussions with the wider research team and PPI member reflections. The extent to which this was *reflexive thematic analysis*, partly rests on the acknowledgement of the researcher’s role in knowledge production. Ongoing reflexive engagement in beliefs, background and assumptions, development as a researcher, and how each of these influenced data analyses were key in the analytical work required.^22^

#### Ethical issues

Ethical approval was obtained for the study from the London- Bloomsbury Research Ethics Committee (REC reference 21/LO/0591) on 17th November 2021. Informed consent was provided by participants and monitored throughout interviews. If any participants became distressed during interview, taking a break, pausing or stopping the interview was suggested. Follow-up telephone calls were offered to participants 24–48 h after interview to check whether they had any questions or concerns. Participants were encouraged to seek support from an appropriate professional such as a member of staff at the children’s hospice from which they were recruited for the study or their GP/trusted healthcare professional should they have needed to.

#### PPI

A family advisory board made of up parents of children with a life-limiting condition contributed to the study at all stages including the study design and developing and piloting the survey and topic guide (including content, structure and pace of interviews). Member reflections also contributed to theme development.^12^ A key suggestion made by the group was to use the survey as a recruitment tool for the optional interview to add flexibility in how fathers took part. The fathers in the group helped to refine the wording of the information sheets, study adverts and survey questions and described the length of the survey as being acceptable (once a progress bar had been added). Contact with the fathers was made at the PPI meetings (two meetings during study/protocol development and one during data analysis/write up) and via email/telephone where appropriate for piloting the survey and topic guide.

## Results

### Survey

#### Sample characteristics

Thirty-two fathers (aged 36–54 years) took part in the survey. Most fathers described their ethnicity as White (94%) and most were born in the UK (94%). The majority described themselves as either their child’s primary caregiver (*n* = 16) or as having shared caregiving responsibilities with their partner (*n* = 9; Table 2).

**Table 2.** Participant characteristics.

Participant characteristics (*n* = 32)	n (%)
Age (range)	36–54 years
Ethnic group	*n* (%)
English/Welsh/Scottish/Northern Irish/ British)	30 (94)
Other ethnic minority group	2 (6)
Location in the UK	*n* (%)
East of England	5 (16)
East Midlands	10 (31)
North-East	2 (6)
London	9 (28)
Yorkshire and the Humber	4 (13)
South West	2 (6)
Religion	*n* (%)
Christian	11 (34)
Jewish	1 (3)
Muslim	2 (6)
No religion	18 (56)
Highest qualification	*n* (%)
GCSE	6 (19)
A-Level	7 (22)
Bachelor’s degree	11 (34)
Master’s degree/PhD	7 (22)
Vocational qualification	1 (3)
Employment status	*n* (%)
Home/caring duties 12 (37)	12 (37)
Full-time work 15 (47)	15 (47)
Part-time or casual work 5 (16)	5 (16)
Employment change following child’s diagnosis	*n* (%)
Stopped work to care for child	11 (34)
Reduced hours to care for child	9 (28)
Change in career path	6 (19)
No change in career path	6 (19)
Caregiving role	n (%)
Child’s primary caregiver	16 (50)
Joint caregiving responsibilities	9 (28)
Child’s other parent is their primary caregiver	7 (22)
Household income per year before tax	*n* (%)
<£10,000	1 (3)
£10,000-24,999	11 (34)
£25,000-49,000	6 (19)
£50,000-£74,999	4 (13)
£75,000–99,000	7 (22)
£100,000 or more	3 (9)
Relationship	*n* (%)
Married or domestic partner	29 (91)
Separated	2 (6)
Single, never married	1 (3)
Number of children	*n* (%)
1	12 (37)
2	13 (41)
3+	7 (22)

Thirty-two fathers of 38 children, aged 3–23, took part in the survey. Most children had been diagnosed with a neurological (*n* = 12) or genetic (*n* = 17) condition, and most had multiple diagnoses (*n* = 26). Thirsty-four children had diagnoses that meant they required significant care, most or all of the time, compared with children of a similar age. The majority of children received care from a children’s hospice (Table 3).

**Table 3.** Child characteristics.

Child characteristics (*n* = 38)	
Life-limiting condition diagnostic category	*n* (%)
Cardiac	3 (7.9)
Congenital	4 (10.5)
Genetic	16 (42.1)
Metabolic	1 (2.6)
Neurological	12 (31.6)
Unknown or no formal diagnosis	2 (5.3)
Age	
Range (years)	3–23
Mean age (years)	11.7
Sex	*n* (%)
Male	18 (47.4)
Female	20 (52.6)
Age at diagnosis	*n* (%)
Before birth	2 (5.3)
At birth	4 (10.5)
Infancy (0–1 years)	9 (23.7)
Childhood (1–9 years)	21 (55.3)
Unknown	2 (5.3)
Needs relative to other children of similar age	*n* (%)
Significantly more care; most or all of the time	34 (89.5)
Moderately more care most of the time	4 (10.5)
Children’s hospice user	*n* (%)
Yes	28 (73.7)
No	10 (26.3)

Table 4 shows the nature of health problems described by fathers, their sleep disturbance scores and caregiving appraisal scores.

**Table 4.** Summary of fathers’ health, sleep and caregiving appraisal data.

Physical or mental health concerns	*n* (%)	
Yes	18 (56.3)	
No	14 (43.7)	
Nature of concern reported	*n* (%)	
Mental health problems	13 (40.6)	
Arthritis	2 (6.3)	
High blood pressure	4 (12.6)	
Back problems	5 (16.6)	
Other illness or progressive disability	1 (3.1)	
PROMIS sleep disturbance^a^		
Mean (SD), range	29.06 (7.81), 10–40	
T-score mean (SD), range	61.66 (9.03), 38.1–77.5	
UK reference T-score^23^	51.26 (8.97)	
Appraisal of caregiving (FACQ-PC)^b^	Mean ± SD	Comparison parent data^15^ Mean ± SD
Caregiver strain	3.9 ± 1.1	3.9 ± 0.7
Positive appraisals	3.7 ± 1.1	4.2 ± 0.7
Caregiver distress	3.5 ± 0.9	3.3 ± 1.0
Family well-being	4.0 ± 0.9	3.6 ± 0.7

a Raw summary scores from the PROMIS sleep disturbance scale were used to describe general sleep disturbance. Each item in the scale is rated on a 5-point scale, meaning that total raw scores can range between 8 and 40, with a higher score indicating higher sleep disturbance. These scores were converted to a standardised T-score (with a mean of 50 and SD of 10) based on recommended scoring.

b The mean scores for each subscale were calculated (range 1–5) with higher scores indicating higher levels of the construct being measured.

Health related quality of life for the five dimensions of the EQ-5D-5L (mobility, self- care, usual activities, pain/discomfort and anxiety/depression), VAS and summary score are shown in Table 5. The mean index score for the sample was 0.89 (1 = best possible QoL). Participants were also asked to rate their health on the EQ-VAS scale. The mean EQ-VAS score was 71.91 (0 = the worst health you can imagine and 100 = the best health you can imagine). Both scores were similar to UK reference scores.

**Table 5.** EQ-5D-5L results for each domain.

EQ-5D-5L results^a^					
	Level 1 *n* (%)	Level 2 *n* (%)	Level 3 *n* (%)	Level 4 *n* (%)	Level 5 *n* (%)
Mobility	30 (93.8)	2 (6.2)	0	0	0
Self-care	32 (100)	0	0	0	0
Usual activities	31 (96.9)	1 (3.1)	0	0	0
Pain/ discomfort	24 (75.0)	6 (18.8)	2 (6.2)	0	0
Anxiety/ depression	8 (25.0)	8 (25.0)	14 (43.8)	1 (3.1)	1 (3.1)
VAS score (mean, SD)			71.91 (13.45)		
Index value mean (95% CI) [range]	0.89 (0.86–0.92) [0.648–1.000]				
UK reference index value mean (95% CI) [range]	0.82 (0.80–0.83) [−0.573 to 1.000]				
UK reference VAS score mean (SD)	71.63 (21.21)				

a Each participant provided a 1-digit number between 1 and 5 for each dimension (no problems = 1 to extreme problems = 5). The digits for each dimension were combined for each participant to describe their 5-digit health state (where 11,111 = full health and 55,555 = worse than death). Health states were then converted into a single index value using a UK specific value set (EuroQol).

### Qualitative analysis

Twelve fathers of 15 children with a life-limiting condition completed interviews, nine of which were initially recruited to the survey via children’s hospices, two via an NHS children’s hospital and one via social media. The fathers that took part in the interviews were aged between 39 and 51 years and resided across various regions on the UK. Most fathers were in full-time (*n* = 7) or part-time employment (*n* = 1), and the rest (*n* = 4) were full-time caregivers. The majority of the sample were White, and one participant was from a minority ethnic group. Their children were aged between 3 and 23 years, 7 were male and 8 were female. Their children had a range of diagnoses including congenital (*n* = 2), genetic (*n* = 5), neurological (*n* = 6), metabolic (*n* = 1), and cardiac conditions (*n* = 1). Interview length ranged from 32 to 88 min. The average interview length was 61 min.

Thematic analysis generated three main themes. The first surrounds the precarious nature of fathers’ day-to-day lives. It focusses on the impact of, and challenges associated with, maintaining uncompromising care routines for their child, creating an overarching, multidimensional sense of precarity. The second surrounds fathers’ navigation of distress; their experiences of unaddressed past traumas, alongside ongoing present day distressing experiences, and the anticipation of future distress or trauma. The third focusses on the scope and severity of the impacts of caregiving on fathers, and a lack of understanding of this from others around this, particularly from healthcare professionals.

#### Theme 1: Everyday precarity

Precarity framed fathers’ experiences. It describes the inflexible, uncertain, and unstable nature of fathers’ day-to-day lives; the intersecting and multidimensional sources of uncertainty that left fathers living in a precarious situation. Fathers, for example, did their best to maintain uncompromising care routines for their child; the non-negotiable responsibilities as part of a closed and prioritised system, leaving little time for anything else. Social activities, work, family time and hobbies were on the periphery. One father described the precision and care with which it took to get his child to appointments.

Getting her out of the house is really tricky and really hard work. You’ve got to pre-heat the car to make sure it’s the same temperature as the room she was in. You have to run her out of the house like a rugby ball, through the outside and into the car. You have to hope that whatever room she’s going into after the car is about the same temperature. Any failure in this will cause a seizure (father 10).

Maintaining high levels of control to maintain strict routines, relied on things ‘going right’. The possibility that they may not, generated fragility. The threat of collapse of these routines was mainly due to the inherent instability and vulnerability associated with the child’s condition itself.

Last year she was in hospital seven times from September to March, and basically she’d get a cold or a little bug, but when she gets a cold she’ll throw up and because then when she throws up she gets dehydrated she has to go to hospital to get rehydrated. So almost on a three week. . . she’d be well for a week, then go to hospital for a week, and that was kind of all last winter. Last winter was probably one of the worst, apart from when she was born’(father 4).

Maintaining the required care routines often relied upon daytime or overnight carers for their child though fathers worried about reductions in, or the complete withdrawal of, this type of support, adding to the sense of living precariously.

‘We used to survive on three or four hours of sleep per night’, which was just awful. Now, we get better sleep but that’s all changing again because his carer that he’s had for three years has just quit and left (father 6).

The nature of precarity was multi-dimensional and required emotional labour as the inherently precarious nature of life-limiting conditions and care provision interacted with more peripheral factors, such as the fathers’ ‘normal’ life events and activities. Fathers experienced precarity not only in relation to the practicalities and logistics of being caregivers, but in their own emotional and psychological states. They worried about additional stressors in other aspects of their lives and that they would not be able to cope with extra uncertainty or challenges or that such would impact their ability to uphold their responsibilities in maintaining their child’s care routine. For example, one father described his flexible and well-paid job as enabling him and his partner to manage their caregiving responsibilities but highlighted his concerns should this change.

*I*’*m well aware that it*’*s on me. I do okay money wise but if I lost my job tomorrow things would quickly become very difficult. It*’*s a lot to manage stress wise. . . knowing that the security of my family, the house, everything, is down to me*’(father 6).

For working fathers especially, there was a real inflexibility of routine and an overwhelming sense of constant responsibility whether that be caregiving or to their employer.

So, I get into the office for 9am, I leave at 7.20am. The bus comes to pick [child] up at 8.30am and [sibling] gets the bus to school too. So anyway, up until 9am it’s quite eventful, even more so if she’s had seizures in the night because then we’re all tired. The school bus brings [child] home for 4.30pm and I look after her while my wife prepares tea. [Child] is put on a feed for teatime and we have meals together. At around 7pm we put [child] to bed. It takes about an hour’(father 2).

Worries about additional stressors went beyond those associated with work. One father described his concerns about his parents needing extra help in their old age, but worried that he wouldn’t be able to provide the care they needed because of the care he was already providing for his child.

My mum and dad are getting on a bit too. . . I just can’t be there as much as I need to be. There isn’t enough time and I just don’t have it in me. . . which worries me as there’s no one else (father 6).

#### Theme 2: Cumulative distress; past, present, and future

**Figure 1.**
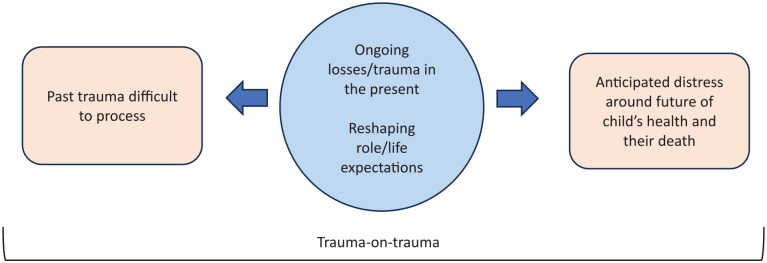
Theme 2 Distress; past, present and future.

The second theme surrounds fathers’ experiences in relation to the course of their child’s illness; the impact of past traumas, ongoing losses and anticipated distress on their current state of wellbeing. These difficult experiences were described as ‘trauma-on-trauma’ with little time to navigate or address such given fathers’ constant caregiving (and other) responsibilities. This theme is depicted in Figure 1 below.

##### Past trauma

For some fathers it had been many years since their child’s diagnosis. For others a shorter period had passed. Regardless of this, there was clarity in fathers’ accounts surrounding the diagnosis itself. They described the traumatic nature of this experience, emphasised by the vividness with which they recounted it. Many fathers had waited for some time for their child’s diagnosis. Fathers described this period as one of uncertainty and ongoing attempts at self-reassurance that nothing was wrong, juxtaposed by readjusting these hopes at various stages, based on new information.

We took her to A&E and they thought it was some kind of wind, some kind of gastronomic issue. So she was put on a ward for those issues. They monitored her there and they were beginning to get concerns that it wasn’t wind. They did a brain scan and I wasn’t there because I was working and I thought “oh it’s just a general procedure to rule things out”. Anyway, the next day the report came back and the consultant. . . [sobbing] sorry. From there we knew there was something seriously wrong neurologically’(father 2).

The shock associated with diagnosis was particularly intense for fathers of children diagnosed during the COVID-19 pandemic. There was little to no immediate support following on from diagnosis. They described a brutality in being told their child was ill and felt abandoned. Fathers were given the ‘worst news of their lives’ and then ‘just left to get on with it with no clue of how to do that’. Fathers felt isolated and commented on how this set a precedent for their expectations of support from professionals throughout the course of their child’s illness.

We were copied into a medical letter. They basically sent us a letter saying “there is something seriously wrong with your child”. There was no compassion in it at all. We were so upset that it was broken to us in this brutal way’(father 7).

##### Ongoing losses and distress

Fathers experienced a deep sense of loss related to opportunities for their child, in addition to the life that they had envisioned for themselves as fathers and uncertainty related to their roles. They described these types of losses as ongoing, often in relation to seeing the pace of development of other children.

We used to take her to baby groups and it was just so disheartening to see other children developing and she just obviously wasn’t’(father 2).

One father described the changes to his child’s communication.

She used to enjoy being read to, now she doesn’t. She lost recognising her cuddly toy that she goes to bed with, she’s lost recognising her name. She used to be able to recognise my wife and I when we came into the room, but she doesn’t anymore. I mean she knows who we are, but she doesn’t greet us in the same way. One thing that I found particularly sad, is that she has stopped smiling. It has been a struggle’(father 10).

##### Anticipating suffering

The final aspect of this theme deals with the anticipated distress that fathers described in relation to their child’s future and decline in condition, including their death. The hypervigilance that fathers described in relation to their child’s symptoms resulted from an awareness that their child’s condition could deteriorate at any moment and was very much built on those experiences of sudden crisis. Fathers highlighted the constant presence of their child’s mortality, alongside inherent uncertainty and fear, particularly when their child needed to be admitted to hospital.

It’s constant, absolutely constant and the thing which I always find, this is when I get quite tearful, that with all this stuff you’re constantly thinking about when [child] is going to die. When she could die. That’s always there in the background. She’s completely healthy now but you’re always thinking she could die. This could be the moment when she doesn’t come out of this, which could be a really long and painful process in itself. She won’t just drop down dead. It will be long and drawn out’(father 4).

For some fathers, the concept of their child’s death was too hard to make sense of. They wanted to know as little about the future of their child’s condition as possible. This conflicted with fathers’ desire to reduce uncertainty and be prepared and informed about their child’s condition.

I’ve tried that, it’s pointless; we’re very aware of the problem and we’re very aware that there isn’t a happy ending. So, great what now?’(father 7).

##### ‘Trauma on trauma’

Fathers described the cumulative impact of these distressing experiences. Fathers felt that many of their experiences remained unresolved alongside not knowing how to manage anticipatory distress. They had little time or understanding of how to process all of this alongside trying to maintain a sense of control over the everyday. Subsequent challenges became harder to navigate emotionally and psychologically.

I think the key thing is that in that 5 months in hospital we never had the chance to process what had happened because you just try to get from one day to the next. She was in intensive care, she had two respiratory arrests, she had a cardiac arrest, she had three surgeries. You just get through from one day to another and then all of a sudden you’re back living in the real world and it’s quite scary’(father 8).

Another father reiterated the lack of opportunity to process distress.

I think I just need to go back to all the stuff that happened when she was born. It’s all just so intense and traumatic, just the whole thing. I would love to be able to go back and have full- on therapy and go through all of that stuff when she was younger. But because of all the stuff happening now you just don’t have time or space to do that’(father 4).

#### Theme 3: The scope and severity of the impact of caregiving on fathers; a lack of understanding from others

This final theme surrounds a lack of understanding of the impacts of caregiving on fathers, as demonstrated through a lack of appropriate support. Fathers spoke about the impact of caregiving on their health, generated by their experiences, outlined in the previous two themes. They mainly focussed on the impact on their mental health but there was real variation in the way they described this, as well as how they managed it. One father described ‘keeping an eye on his mental health’, deciding whether he was feeling understandably stressed or whether his depressive symptoms needed to be addressed through his General Practitioner (GP).

I guess that if I felt as though I was going into a mental health crisis then I would talk to my GP. If that’s the type of thing I needed, I could progress that but it doesn’t seem like a pressing concern at the minute’(father 10).

This ad-hoc type approach to support seeking was helpful and complimented their ‘getting on with it’ approach to caregiving. Conversely, it meant that fathers could be faced with unclear thresholds for support, ‘putting up’ with things and leaving concerns unaddressed until they became urgent and/or overwhelming.

I think I pushed on and pushed on and pushed on to the point where I just couldn’t keep going, it was like a fuse had blown’(father 5).

Much of this was rooted in gendered assumptions of health and support seeking.

Us dads don’t talk. I know an awful lot of dads out there that don’t because you always feel that you’re expected to be strong. As a dad, it’s your job to protect your child and your entire family. I think a lot of dads struggle with that’(father 8).

For those without pre-established relationships with professionals, or routes to support, there was little appropriate help available. Fathers described their experiences of support with particular reference to interventions that lacked the ability to understand or address their needs.

The best she could come up with was some deep breathing exercises. The following week she was telling me about therapeutic doodling, just get a pen and paper and doodle for a bit. I was like I’m not entirely sure that’s going to answer my questions’ (father 1).

As well as highlighting inadequacies in addressing their needs, there were also safety concerns surrounding short-term psychological interventions aimed at supporting fathers’ mental health.

I found it quite painful honestly and that the six to eight sessions available, that it wasn’t really safe in the scope of such a short period to go into the depths of what was going on and also in the time after the sessions. I would be less functionally available to the family because of what I had to go into and come out of’(father 10).

Although the majority of fathers’ experiences surrounded a lack of understanding from healthcare professionals, there was also a lack of understanding from other people. Fathers described their relationships with friends and family as challenging, due to their lack of ability to understand what fathers were going through/what their needs might be.

Sometimes you’ve spoken to family members, they’d say things like “Oh I know somebody who had epilepsy and they stopped having it when they were 11”, which to be fair is an ordinary persons experience of it. I think people just don’t see it, always looking for solutions or to reassure me that things are going to be okay. But you know it ain’t going to happen’(father 2).

## Discussion

### Main findings

Fathers experienced various forms of precarity in their everyday lives. This precarity often existed as a direct result of the inherent instability of their child’s condition. Fathers were ‘at capacity’, both physically and emotionally, making it difficult for them to deal with other life stressors. The importance of understanding fathers’ experiences over the course of their child’s illness was highlighted through the second theme surrounding repeated and/or unresolved distress alongside anticipated trauma. Fathers felt practitioners lacked understanding around what caregiving for a medically complex child entailed, as demonstrated through inappropriate support offered. They highlighted the inherent failure of short-course therapies in offering long-term and more sustainable support. This was particularly prominent in fathers’ accounts as they mentioned that more, or less, support may be needed at various points over the course of their child’s illness.

### Strengths and limitations

The semi-structured, in-depth qualitative interviews were able to provide rich data on the health and caregiving experiences of fathers. Studies have rarely asked fathers about their health directly, meaning that this study provides an important contribution to how we understand the impacts of caregiving and a foundation for future research aimed at exploring tailored support for parent caregivers. The small sample size for the quantitative component meant that it was only feasible to provide descriptive results. Therefore, there were implications on the nature of integration with the qualitative data, which would have perhaps provided deeper insight had there been the opportunity for more scope in the analysis of the quantitative data. The survey was still able to provide important contextual information about fathers, as well as providing them with the opportunity to engage with the topics that would be discussed in the interviews. There was a lack of ethnic diversity in the sample of fathers which is particularly important to address in the next phase of this research given the prevalence of life-limiting conditions in ethnic minority groups.^1^ Further work needs to be carried out surrounding engagement and recruitment strategies that encourage diverse participation, and that go beyond recruiting ‘fathers’.

### What this study adds

Uncertainty has long been established as an important construct in parental caregiving,^24^ though the multi-dimensional and pervasive sense of everyday precarity in fathers’ accounts goes beyond what is reflected in existing studies. In this study, the precarity experienced by fathers in relation to their child’s condition was heightened by the fragility of the support systems in place for their child. ‘Precarity’ is often used as a rather inflexible framework for understanding the impact of insecure labour conditions.^25^ However, its application more generally allows us to explore and contextualise broader uncertainties, capturing lived experiences of precarity, as opposed to precarity as a social condition.^26^ The term is being increasingly used in health research, particularly in understanding illness experiences and uncertainties of older adults and their caregivers.^27,28^

The cumulative nature of the distress experienced by fathers throughout the course of their child’s illness was captured by the concept of trauma-on-trauma. Recent research highlights the need for trauma-informed palliative care^29^ though current evidence surrounding suitable approaches are limited. The majority of what we understand about trauma and parental mental health comes from literature surrounding paediatric oncology populations,^30^ meaning that we know little about how this trauma may manifest in fathers of children with life-limiting conditions with very different trajectories. Supporting fathers across these trajectories, requires greater recognition of the nuance involved in this type of caregiving, particularly the parent-child relationship and the impact of feeling unable to play a protective role for their child.^31^ The anticipation of their child’s condition deteriorating, and indeed their death, is important to consider alongside how fathers are supported.

Singer et al. proposed the concept of illness-related grief encapsulating present day losses related to illness.^32^ Understanding forms of grief is helpful as a means of identifying targets for support and interventions, and useful in delineating different types of trauma and the need for support for loss and grief across the course of a child’s illness.^33^

For children and families who have access to paediatric palliative care services, there will often be grief support available long before the child’s death. However, geographical differences mean that there is significant inequity in availability and access to support for individual children.^34^ Issues such as delays in referrals also mean that children with the most complex needs and their families do not have access to specialist services that can provide such support.^35^ As shown, not only do fathers’ experiences and needs change over the course of their child’s illness, but vary across individuals. The lack of appropriate support is very much similar to what has been described by mothers.^6^ This study suggests that time spent caring, parental roles and/or caregiving roles may not be predictive of support needs.

## Conclusions

The precarity in fathers’ accounts demonstrates the constant sense of insecurity and vulnerability that fathers experienced. This goes further than ‘caregiver uncertainty’, as it explores the impact of cumulative uncertainties; the fragilities and unpredictability inherent to their child’s condition and care, as well as those associated with the broader aspects of fathers’ lives. Understanding the impact of various forms of precarity reiterates difficulties in disentangling parental experiences from the needs of their child. Support tailored to these parents should account for this. There are important differences between parental distress because a child is not getting appropriate care and support, and distress because the parent is not being well supported themselves.

The way in which the literature currently conceptualises trauma in relation to childhood injury and illness may be useful, though it does not fully incorporate important considerations of temporality. Fathers’ needs will fluctuate over the course of their child’s illness, demonstrated across themes, but there are key timepoints for professionals to be mindful of in the trajectory of the child’s illness and in the development of trauma-informed palliative care.^29^ This has not yet been fully explored, particularly in terms of how fathers’ past experiences, in relation to their child and their own health, shape future expectations and perspectives, as well as their ability to cope with stress. Current mechanisms by which fathers’ support needs can be identified and addressed do not sufficiently capture important aspects of their experiences suggesting that there is a real need for tailored interventions able to do so.

## Supplemental Material

sj-docx-1-pmj-10.1177_02692163251327877 – Supplemental material for A mixed methods exploration of the health and caregiving experiences of fathers of children with a life-limiting condition Supplemental material, sj-docx-1-pmj-10.1177_02692163251327877 for A mixed methods exploration of the health and caregiving experiences of fathers of children with a life-limiting condition by Victoria Fisher, Karl Atkin and Lorna K Fraser in Palliative Medicine
