# Supplementary material for: A mixed methods exploration of the health and caregiving experiences of fathers of children with a life-limiting condition
Source: Palliat Med. 2025 Mar 25;39(6):678–88. doi: 10.1177/02692163251327877 (PMC12102508; doi:10.1177/02692163251327877)
Supplement: sj-docx-1-pmj-10.1177_02692163251327877 – Supplemental material for A mixed methods exploration of the health and caregiving experiences of fathers of children with a life-limiting condition [file sj-docx-1-pmj-10.1177_02692163251327877.docx]

**Research Study: The health of fathers of children with a life-limiting condition; a mixed methods study**

Topic guide for interviews with fathers

1. Introducing the interview and consenting process

- Researcher introduces self
- Explain/reiterate: confidentiality, length of interview, nature of discussion, explain that participant can withdraw at any time, fine to take breaks
- Does the participant have any questions
- Obtain consent
- Start recording

2. Finding out about the father and their family

- Example opening scripts: “To start off, please could you tell me a little bit about you and your family?...”
- Probes:
- number of children
- children’s ages and family composition
- what a typical day is like including experience of caregiving
- what is your experience of supporting your children?
- what were your feelings when you first found out there was something wrong with one of your children?

3. Caregiving

- Exploration of fathers’ roles as caregivers.
- Probes:
- Employment
- Roles in caring for child and other children
- How/ if role has changed
- Thoughts/ feeling associated with caregiving/ role

. Fathers’ health and wellbeing

- Example opening script: “Now if we could move onto talking a little bit about your own health now, is that okay?”
- Probes:
- Any concerns with regards to your physical or mental health/ how would you describe your own health?
- Particular factors that fathers feel may have harmed or have helped/ protected their health
- Means of support and ease of access; formal or informal
- Experiences with healthcare professionals
- When was the last time fathers were asked about their own well-being/ have fathers been made to feel like their own health is important?

5. Follow-up questions on target topic areas:

- Views on how easy it is to access healthcare for themselves
- Workplace support
- Relationships with healthcare staff
- Views on how health has changed over time and in relation to their child’s illness
- Coping

6. Close:

- Express thanks and note moving towards end of interview
- Ask if anything else they would like to add
- Remind re confidentiality, next steps in research and when findings will be ready
- Check if any questions
- Arrange follow-up contact
- Reiterate thanks and appreciation for time/ taking part
